# Characterization of halo-tolerant plant growth promoting endophytic *Bacillus licheniformis* MHN 12

**DOI:** 10.1186/s43141-022-00407-3

**Published:** 2022-08-03

**Authors:** Namita Bhutani, Rajat Maheshwari, Nidhi Sharma, Pradeep Kumar, Amita Suneja Dang, Pooja Suneja

**Affiliations:** 1 Department of Microbiology, Maharshi Dayanand University, Rohtak, Haryana 124001 India; 2 Centre for Medical Biotechnology, Maharshi Dayanand University, Rohtak, Haryana 124001 India

**Keywords:** Nodules, Plant growth promoting endophytic bacteria, Salt stress, TEM, *Vigna radiata*

## Abstract

**Background:**

Endophytic bacteria overlay significant role in plant growth promotion, eliminating phyto-pathogens and combating stress-conditions. In the present study, we aimed to screen high salt tolerant bacteria and study their adaptive response to elevated salt concentrations. A total of 46 endophytic bacterial isolates from *Vigna radiata* were screened for salt tolerance. The high salt tolerant endophytic isolate was characterized for alteration in morphology, growth rate, protein profiling, and compatible solute concentrations.

**Results:**

The isolate MHN12, based upon biochemical characterization and partial 16S rDNA sequencing identified as *B. licheniformis* (accession number MG273753) was able to tolerate up to 15% NaCl (Sodium Chloride) (2.6 M) concentration. The isolate possessed 1-aminocyclopropane-1-carboxylic acid deaminase (ACCD) activity along with indole acetic acid (IAA), siderophore, ammonia, organic acid and hydrogen cyanide (HCN) production. Accumulation of proline was apparent up to 7.5% NaCl concentration and declined afterwards. Ultrastructure analysis using TEM (transmission electron microscopy) revealed the morphological alteration from rods to filaments.

**Conclusion:**

Acclimatization to salt stress and plant growth promoting activities could contribute to utilization of this bacterium as bioinoculant to enhance the crop yield and discourage the application of chemical fertilizers.

## Background

Globally, agricultural practices focus mainly on the scale of production instead of environmental safety. Insertion of chemical fertilizers on a large scale has created a pollution hazard and loss of soil productivity [1]. Industrialization has also taken a toll on the soil quality, mostly by the release of effluents into land, thereby seeping deep into the ground water which in turn makes the soil saline and posing a threat to soil fertility. The salinization of cultivable land is increasing and presenting a challenge to agriculture worldwide [2]. To deal with soil salinity, various strategies like resource management, improving salt tolerance of plants either by breeding or transgenic plants are employed. Being time consuming and cost intensive, need arises to explore simple and low-priced methods in order to benefit the agriculturist.

In addition to traditional and modern breeding practices, the bad effects of salinity can be overcome by use of salt tolerant bacteria with natural plant growth promoting traits [3]. Endophytes traditionally defined as the microorganisms either bacteria or fungi which harbour the plant tissues and can be isolated after surface sterilization [4]. Both rhizobial as well as non-rhizobial endophytes reside inside the nutrient rich habitat of roots and nodules of leguminous plants [5]. They are sheltered from microbial competition and environmental stresses by host plant and subsequently enhance the health of their host, indirectly or directly by acquiring nutrients, by fixing nitrogen, by producing siderophores and solubilizing phosphate [6, 7]. Furthermore, some endophytic bacteria deplete the level of plant hormone ethylene increased due to several environmental stresses including salt stress by producing ACC (1-aminocyclopropane-1-carboxylic acid) deaminase [8, 9]. The most commonly reported non-rhizobial endophytic genera in legumes are *Enterobacter*, *Pseudomonas*, *Bacillus*, *Mycobacterium*, *Ensifer*, *Rhizobium*, and *Paenibacillus* [10, 11].

Halotolerant endophytes with multiple plant growth promoting traits could play significant role as inoculants as a low cost biological method to combat salt stress. Numerous investigations on the interaction of PGPR (plant growth promoting rhizobacteria) with plants growing in saline habitats are still at a budding stage [12, 13]. To adapt themselves in saline conditions bacteria imply either of these two components: ionic component linked with toxicity due to penetration of ions into the cell affecting cellular pH as well as enzyme activity and osmotic component due to increase in solute levels outside the cell [14]. The bacterial cell morphology, chemical composition of membrane, and synthesis pattern of biomolecules such as proteins, lipids, polysaccharides is altered due to salt stress in both salt-sensitive and salt-tolerant bacteria [15]. Due to salt stress, the upregulation or downregulation of some existing proteins as well as synthesis of new proteins has also been reported [16]. Bacteria produce osmolytes which help in the osmoregulation and prevent dehydration of cells due to salt stress. Members of genus *Bacillus*, one of the most commonly found endophytes in plants is widely distributed in soil and have evolved to bear sudden environmental stresses such as nutrient starved and drought conditions, rise in temperature, increase in salinity, etc. [17]. Osmoprotection by uptake of exogenous proline have been reported in various members of genera *Bacillus* [18]. However, morphological adaptations and changes in growth rate at higher salt concentrations are not well studied.

Therefore, it is required to explore the rich sources of nature to find out appropriate endophytic bacteria from native plants, which can alleviate salt stress in plants. In this study, the endophytes were isolated from nodules and roots of *Vigna radiata* and screened for salt tolerance. The salt tolerant endophytic bacteria was molecularly identified and characterized for ACC deaminase activity as well as other plant growth promoting properties. In addition, the cell morphology, multiplication, growth curve, and protein profiling at various salt concentrations and ultrastructure were assessed.

## Methods

### Screening of endophytic bacteria for salt tolerance

The bacterial endophytes isolated from nodules and roots of *Vigna radiata* from Bhiwani (28.41° N: 75.86° E), Hisar (29.15° N: 75.75° E), Charkhi Dadri (28.50° N: 75.93° E), Fatehabad (29.59° N: 75.78° E) districts of Haryana were obtained from plant-microbe interaction laboratory, Department of Microbiology, Maharshi Dayanand University, Rohtak, Haryana, India [19]. All these isolates were evaluated for their salt tolerance ability on tryptone soya agar (TSA) media supplemented with varying NaCl concentrations (2.5%, 5%, 7.5%, 10%, 12.5%, and 15%). After an incubation of 48 h at 28 ± 2 °C, growth pattern of respective isolates was observed. Three replicates of isolates were used for each salt concentration.

### Characterization of selected isolate

#### ACC deaminase activity

The isolate able to grow at 15% NaCl concentration was selected and characterized for the presence of ACCD enzyme. The initially screening was done on DF minimal medium [19] amended with 3 mM ACC as sole nitrogen source. DF medium without ACC and with ammonium sulfate were used as negative and positive control respectively. The ACC deaminase activity was determined by growing the isolate overnight in culture broth. Cell biomass was harvested, washed and suspended in DF media tubes. To induce ACC activity, 45 μl of 0.5 M ACC added to the cell suspension so as to achieve a final conc. of 3 mM and incubated at 200 rpm for 24 h. Cells were again harvested by centrifugation at 4390×*g* for 10 min at 4 °C and washed with 5 ml of 0.1 M TrisHCl (pH 7.6). Again, 1 ml of 0.1 M TrisHCl was added to the pellet and centrifuged at 22938×*g* for 5 min at 4 °C. The pellet obtained was resuspended in 600 μl of 0.1 M TrisHCl (pH 8.5) and 30 μl of toluene and vortexed. Immediately 20 μl of ACC (0.5 M) was added to toluenized cells (200 μl), vortexed and incubated at 30 °C. After 15 min incubation, 1 ml of 0.56 N HCl was added, centrifuged (22938×*g*) for 5 min at 4 °C. The 1 ml supernatant was again vortexed with 0.56 N HCl (800 μl) and 0.2% DNPH solution (300 μl) and incubated at 30 °C. To this 2N NaOH (2 ml) added and absorbance measured at 540 nm. The enzyme activity was checked by comparing it with standard curve of α-ketobutyrate (0.1–2 mM) [20].

### Plant growth promoting (PGP) traits

The isolate was also screened for wide array of PGP properties. For ammonia production, peptone water tubes were prepared and inoculated with bacterial isolate. These tubes were incubated for five days at 28 ± 2 °C. After incubation Nessler’s reagent added and presence of yellow to brownish orange color indicates positive test. The ability to produce organic acid was checked by methyl red test. The bacterial isolate was grown in MR-VP broth. The methyl red was added and presence of bright red color shows the positive reaction [21].

Hydrogen cyanide (HCN) production was screened with the method as described by Lorck [22]. Freshly grown culture broth was spreaded on nutrient agar plates supplemented with glycine and Whatman filter paper (No.1) dipped in 0.5% picric acid was placed on the cap. The change in color of the filter paper form orange to brown after incubation of 4–5 days confirmed the cyanogen production. The bacterial isolate was also examined for phosphate solubilization. The isolates were tested for their ability to solubilize inorganic phosphate. The isolates were grown in TSB (tryptic soya broth) and spotted on Pikovskaya agar medium plates. After an incubation of 4–5 days at 28 ± 2 °C, development of clear halozone around bacterial growth indicating phosphate solubilization was observed [23]. Salkowski colorimetric assay was used to detect the production of phytohormone IAA. YEM broth was supplemented with 0.1 gL^−1^ L-tryptophan, inoculated with bacterial isolate, incubated at 28 ± 2 °C for 5 days. The broth was centrifuged (4390×*g*) for 3 min at 4 °C. The Salkowski reagent was added to the supernatant (1:1 ratio) and incubated for half an hour in dark. Optical density was measured at 530 nm and production of IAA was estimated using IAA standard curve [24]. Two milliliters of un-inoculated YEM (yeast extract mannitol broth) with equal volume of Salkowski reagent was used as negative control. The ability of isolate to promote plant growth was also assessed in vitro by agar plate assay [25]. The seeds of V. radiata were surface sterilized with 0.25% HgCl2, washed with sterilized water and kept on 1% agar media plates. They were allowed to germinate for 24 h. Again these germinated seeds were placed on 1.2% agar plates and inoculated with freshly grown bacterial culture. After incubation of 5 days, results were recorded. The increase in root and shoot length as compared to uninoculated control was measured. The production of siderophore was detected by CAS (Chrome azurol S) agar plate assay [26]. The bacterial cultures were spotted on CAS agar plates and incubated at 28 ± 2 °C for 5 days. Positive results for siderophore production were indicated by change in medium colour (from blue to orange) and characterized quantitatively using CAS liquid assay [27].

The cultures were grown in Siderophore Inducing Medium (SIM) and incubated at a 28±2°C in rotary shaker for 5 days. After centrifugation for 15 min at 4390×*g* at 4 °C, culture supernatant (0.5 ml), CAS solution (0.5 ml), and shuttle solution (2 M 5-sulfosalicyclic acid) (10 μl) were mixed and subsequently incubated for 20 min at room temperature. Optical density was measured at 630 nm.

Units of siderophore production were calculated as

$$\left\{\left(\mathrm{Ar}-\mathrm{As}\right)/\mathrm{Ar}\right\}\times100\%$$

Where Ar = Absorbance of blank containing media, CAS assay solution and shuttle solution;

As = Absorption of test sample containing supernatant, CAS assay solution and shuttle solution at 630 nm.

### Biochemical characterization of salt tolerant isolate

Isolate was primarily examined for growth characteristics and biochemical characterization such as indole, methyl red, Voges-Proskauer and citrate utilization test (IMViC), sulphate, indole and motility test (SIM), catalase etc. using standard protocol [28]. Other biochemical and carbohydrate utilization tests were performed using HiBacillus KB013 Identification kit (Himedia, India) as confirmatory tests for *Bacillus* species. Results were recorded as shown in the manufacturer’s interpretation chart. The extracellular enzymatic production of amylase, cellulase, and protease was evaluated using nutrient agar media supplemented with 1% starch, 0.5% carboxymethyl cellulose and 1% casein respectively [29]. Pectinase production was determined by observing the growth ability on 0.5% pectin agar medium [30].

### Bacterial growth curve at varying salt concentrations

To observe the effect of salinity on growth, the isolate was grown in TSB (*tryptone soya broth*) with steady increase in salt concentrations (0.5%, 2.5%, 5%, 7.5%, 10%, 12.5%, and 15% NaCl). An aliquot of 2 ml was taken from the culture flask at an interval of every 2 h and absorbance was determined at 600 nm (Thermoscientific Genesis 10SUV-VIS) till a stationary phase was achieved. Growth curve was plotted with optical density versus time.

### Protein profiling

The bacterial cells grown in TSB with steady increase in salinity (0.5%, 2.5%, 5%, 7.5%, 10%, 12.5%, and 15%) as well as sudden uplift in salinity (direct transfer from 0.5 to 15%) were harvested from the late log phase by centrifugation at 8960×*g* for 10 min at 4 °C . Cells were washed in MilliQ, resuspended in 100 μl of sample buffer, pelleted down and further lysed at 100 °C for 10 min in water bath. Lysates were centrifuged at 8960×*g* for 10 min at 4 °C and protein concentration was determined by Bradford method [31]. Thirty micrograms of proteins per lane were loaded on 12% polyacrylamide gel containing 1% SDS [32] and electrophoresis was carried out. Gel was stained with Coomasie brilliant blue (CBB) R-250 and photographed on DNr Bio-imaging Systems, MiniBIS (Neve Yamin, Israel).

### Proline estimation

Bacterial cells were grown at each salt concentration up to late log phase and centrifuged at 8960×*g* for 10 min at 4 °C. The pellet was washed with MilliQ water and then extracted overnight in 1 ml of 3% (aq) sulphosalicyclic acid. After removal of protein precipitates and debris by centrifugation, sample was prepared using Chinard’s reagent [33] and glacial acetic acid at boiling temperature for 1 h. Two milliliters of toluene was mixed in the sample plunged in ice and color was extracted for 15 min. Absorbance of colored compound was observed using toluene as blank at 520 nm. Standard curve was prepared using L-Proline (10–100 μg/ml) (Merck Millipore, Germany).

### Light microscopy

One milliliters of cell culture of all salt concentrations (0.5–15% NaCl) was centrifuged (3226×*g*; 3 min; 4 °C), pellet was washed with saline, Gram stained and observed under Magnus light microscope-MLX-B (SP) using AMCap software for imaging.

### Transmission electron microscopy (TEM)

Five hundred microliters of bacterial broth grown at two salt concentrations (0.5% and 15% NaCl) were centrifuged (4390×*g*; 25 °C; 3 min) and the pellets were washed twice with phosphate buffer saline (PBS). Cells were immediately fixed in 2.5% glutaraldehyde and 2% paraformaldehyde in 0.1 M phosphate buffer (pH 7.4) for 24 h at 4 °C. The samples were then rinsed with the same buffer to remove excess fixative and fixed again with secondary fixative, i.e., osmium tetroxide (1% solution). After removal of excess fixative, samples were dehydrated using ascending series of ethanol and embedded into an epoxy resin. The samples were allowed to get saturated with resin at 50 °C overnight and then hardened at 60 °C for 2–3 days. For TEM, semithin block (0.5–1.0 μm) were taken, trimmed into ultrathin sections (0.06–0.09 μm) using PT-PC PowerTome ultramicrotome (Boeckeler, USA) and observed under Tecnai G2 20S-Twin (FEI) transmission electron microscope at 200 kV.

### Statistical analysis

All the experiments were performed in triplicates. The data was analyzed with Student t-test and evaluated for significant differences at *P* values ≤ 0.001, 0.01, and 0.05 using MS Excel.

## Results

**Fig. 1:**
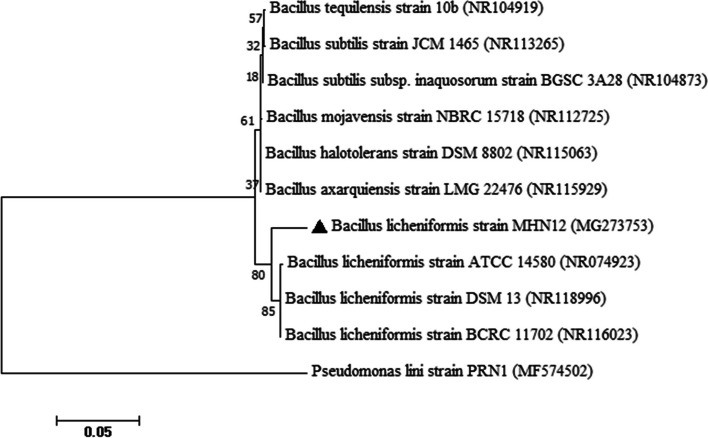
Phylogenetic tree based on 16s rRNA gene sequences of MHN12 with closest related sequences obtained from NCBI

The salt tolerance ability of all the 46 isolates was checked at varying salt concentrations (0.5–15%). All the isolates were growing at 2.5–5% NaCl concentration. While 78% isolates were able to grow at 7.5% and 52% up to 10% NaCl concentration. Growth of 23.9% isolates was observed at 12.5% NaCl concentration, only one isolate MHN12, showed growth at 15% NaCl concentration which was selected for detailed characterization. To ascertain its identity at molecular level, 16S rDNA sequence analysis was done. The maximum likeness of 97% was shown towards *Bacillus licheniformis* strain DSM 13 and *Bacillus licheniformis* strain BCRC 11702. The obtained sequence of the isolate was submitted to GenBank under accession numbers MG273753 as reported [29]. A NJ phylogenetic tree was generated from sequences obtained from NCBI with bootstrap value of 1000 replicates (Fig. 1)

### ACC deaminase activity

Isolate MHN12 was able to utilize ACC as sole nitrogen source on ACC amended DF minimal medium. The enzyme activity was quantitatively assessed in terms of ACC consumed and α-keto butyrate produced. It exhibited specific activity of 24.07 ± 1.79 μmol of α-KB/mg protein/h.

### Other plant growth promoting traits

The isolate showed positive result for IAA and produced 27 ± 0.7 μg/ml of IAA after 5 days of growth. Siderophore was also produced on CAS agar plates by forming orange halo zone and CAS assay revealed production of 90% siderophore units as compared to control. It was also positive for ammonia, HCN, organic acid production, and promoted in vitro root growth (Table 1). However, it was not able to solubilize phosphate as reported [29].

**Table 1:** Plant growth promoting properties of MHN12

PGP trait	Activity
IAA production (μg/ml)	27 ± 0.7
Siderophore (% Siderophore units)	90.09 ± 0.70
ACC deaminase (μmol α-KB/mg protein/h)	24.07 ± 1.79
Phosphate solubilization	−
Ammonia production	+
HCN production	+
Organic acid production	+
In vitro root length^a^ (cm)	8.0 ± 1.3^**^
In vitro shoot length^b^ (cm)	13 ± 1.0^*^

Values are mean of three replicates ± SD

^ns^(Non-significant) indicates *P* > 0.05

*Significant at *P* ≤ 0.05

**Significant at *P* ≤ 0.01

^a^Control (cm) − 3.3 ± 0.58

^b^Control (cm) − 6.3 ± 3.2

### Biochemical characterization and molecular identification of MHN12

The basic microbiological tests revealed that the isolate MHN12 was spore forming Gram positive bacteria. Biochemical tests indicated positive results for Voges-Proskauer, citrate, catalase, nitrate reduction, H_2_S, ONPG, malonate, arginine, and negative for methyl red and indole. The carbohydrate utilization pattern of MHN12 was similar to standard strain of *Bacillus licheniformis.* The isolate was producing extracellular enzymes viz. amylase, protease, cellulase, and pectinase as reported earlier [29] (Table 2).

**Table 2:** Physiological and biochemical characteristics of MHN12

Characteristics	Results	Production	Activity
Isolation source	Nodules	Indole production	Negative
Gram reaction	Positive	Methyl red test	Negative
Cell shape	Rods	Voges-Proskauer test	Positive
Motility	Positive	Citrate utilization	Positive
Endospore	Positive	Catalase	Positive
Colony on TSA	Irregular, cream, rough	H_2_S	Negative
Growth at 10 °C	Negative	Malonate	Positive
Growth at 50 °C	Positive	ONPG	Positive
Growth at 55 °C	Negative	Nitrate reduction	Positive
Common habitat	Soil	Catalase	Positive
**Carbohydrates**	**Utilization**	Arginine	Positive
Sucrose	Positive	**Extracellular enzymes**	**Production**
Mannitol	Positive	Pectinase	Positive
Glucose	Positive	Amylase	Positive
Arabinose	Negative	Protease	Positive
Trehalose	Negative	Cellulase	Positive

### Bacterial growth curve

**Fig. 2:**
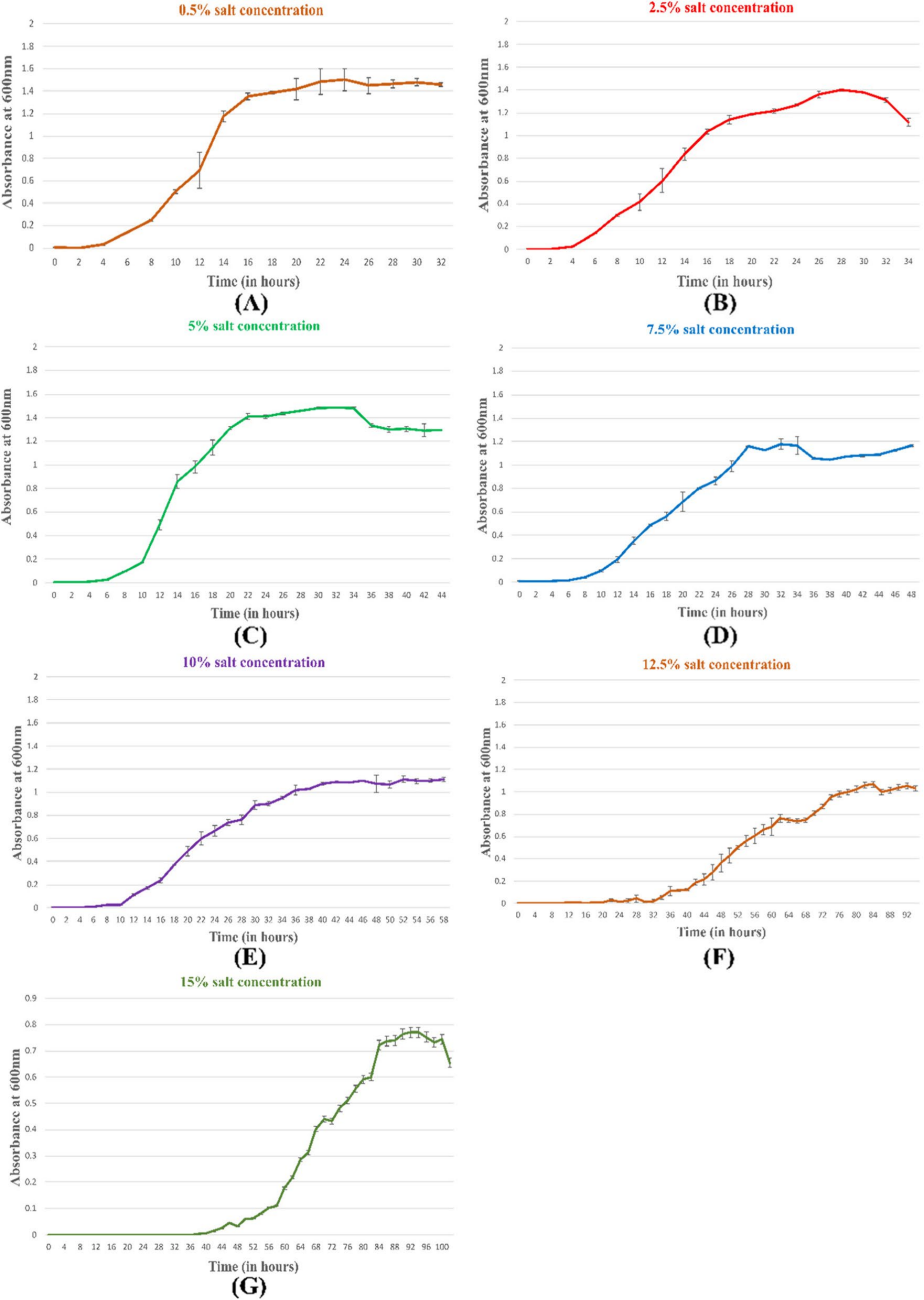
Effect of varying NaCl concentrations on growth of MHN12 grown in TSB incubated at 28 ± 2 °C

Aiming to study the effect of salt on growth curve, MHN12 was inoculated in liquid media at salt concentrations 0.5–15%. As salt concentration was increased, growth gradually slowed down as shown by the increase in duration of lag and exponential phase. The lag phase increased from 2 h in 0.5% and 2.5% NaCl concentration to 44 h in 15% NaCl concentration (Fig. 2a–g). Exponential phase was of around 18 h at low salt concentrations (0.5 and 2.5% NaCl) and reached up to 52 h at high salt concentration (15% NaCl) along with altered morphology.

### Protein profiling

**Fig. 3:**
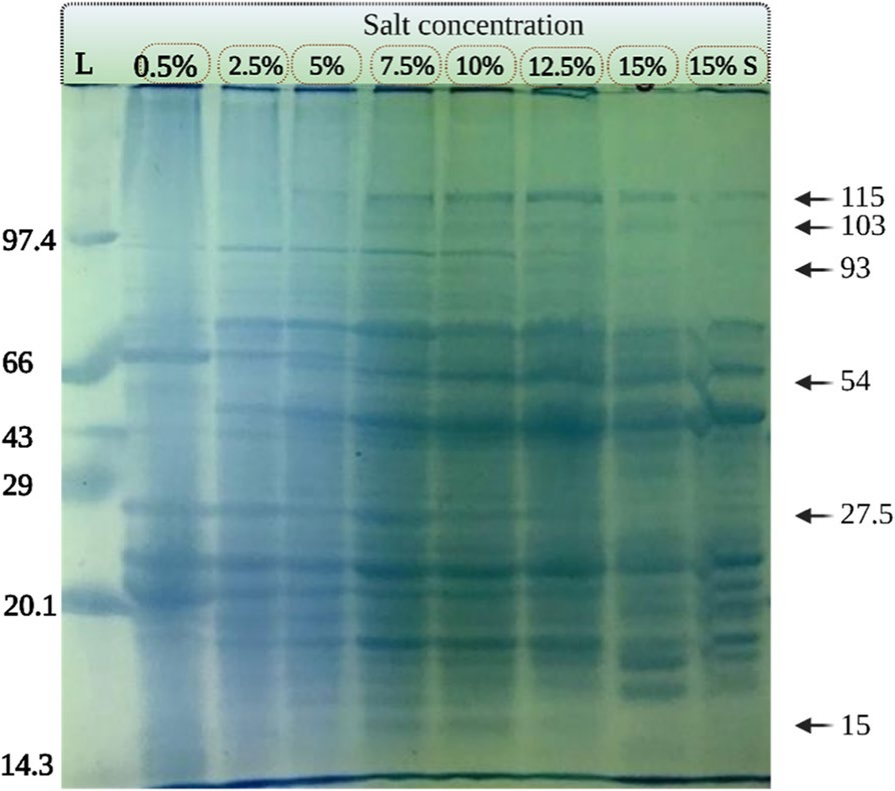
Stained SDS-PAGE of whole cell proteins from MHN12 at varying salt concentrations. Lanes depicts the increasing salt concentration from 0.5 to 15%, 15% shock where L stands for Genei medium range protein ladder

The electrophoretic protein pattern of isolate MHN12 was studied for visualising the changes in protein profile under increasing salt concentrations (0.5% to 15%) as well as under rapid shock treatment. The SDS-PAGE (Sodium dodecyl-sulfate polyacrylamide gel electrophoresis) revealed the variation of many polypeptides at different salt concentrations, among which 6 bands were prominent (Fig. 3). Three bands of 115, 103, and 54 kDa appeared at higher salt concentrations and three bands of 93, 27.5, and 15 kDa disappeared from samples above 10% NaCl concentration. Comparative analysis of lanes showed the over and under expression of some polypeptides. Three bands of 72, 44.5, and 17.5 kDa were overexpressed at 7.5% to 15% salt concentration as compared to the lower salt concentrations and 2 bands of 16.5 and 16.1 kDa were overexpressed at 15 and 15% shock condition only. Underexpression of polypeptides of 20.5 was also observed at higher concentration (15% and 15% shock condition).

### Proline estimation

**Fig. 4:**
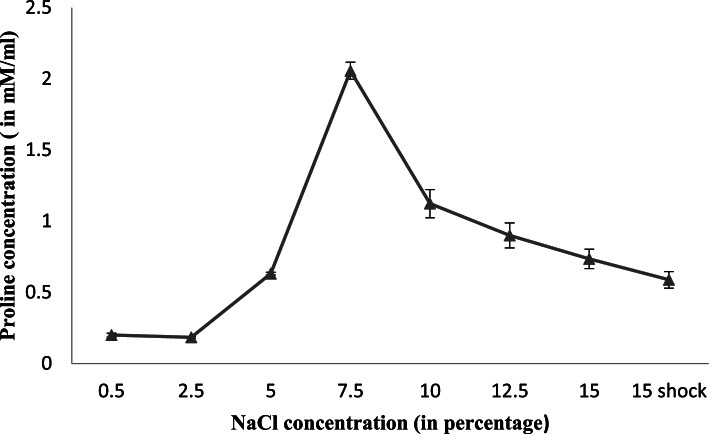
Estimation of total proline accumulation (mM/ml) at varying NaCl concentrations (0.5–15%)

The intracellular proline pool at varying salt concentration was also determined (Fig. 3). An increase in salinity up to 2.5% had no effect on proline content but further rise (up to 7.5% NaCl) in it resulted in accumulation of proline around two folds as compared to control cells. However, at higher salinity level (> 10% NaCl), proline accumulation gradually declined (Fig. 4).

### Light microscopy

**Fig. 5:**
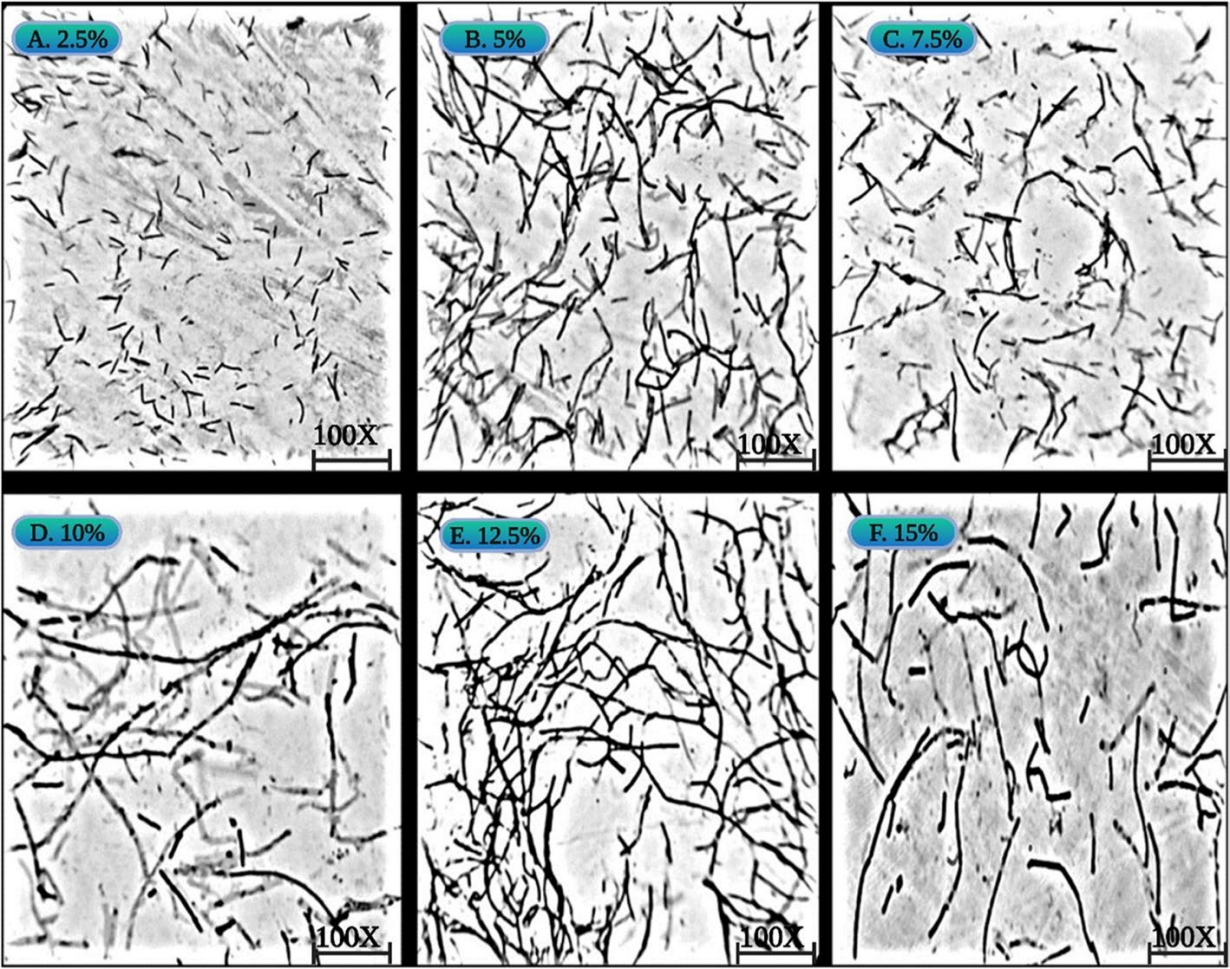
Effect of salt stress on cell morphology of MHN12 at varying NaCl concentration. **a**–**f** 2.5%, 5%, 7.5%, 10, 12.5%, and 15%, respectively

We also evaluated the morphological changes in the test isolate in response to salt stress. Light microscopy imaging demonstrated that there was no change in morphology at 0.5% and 2.5% NaCl concentrations. Strong morphological changes were observed as the salt concentration increased from 5 to 15% NaCl. The structural organization of cell changed apparently from small (0.5–2.5%) to long rods (5–7.5%) to filamentous form (10%, 12.5%, and 15%) with increasing salt concentrations (Fig. 5a–f). The cells were able to repopulate in post stress condition and their morphology again changed from filamentous form to small rods.

### Transmission electron microscopy (TEM)

**Fig. 6:**
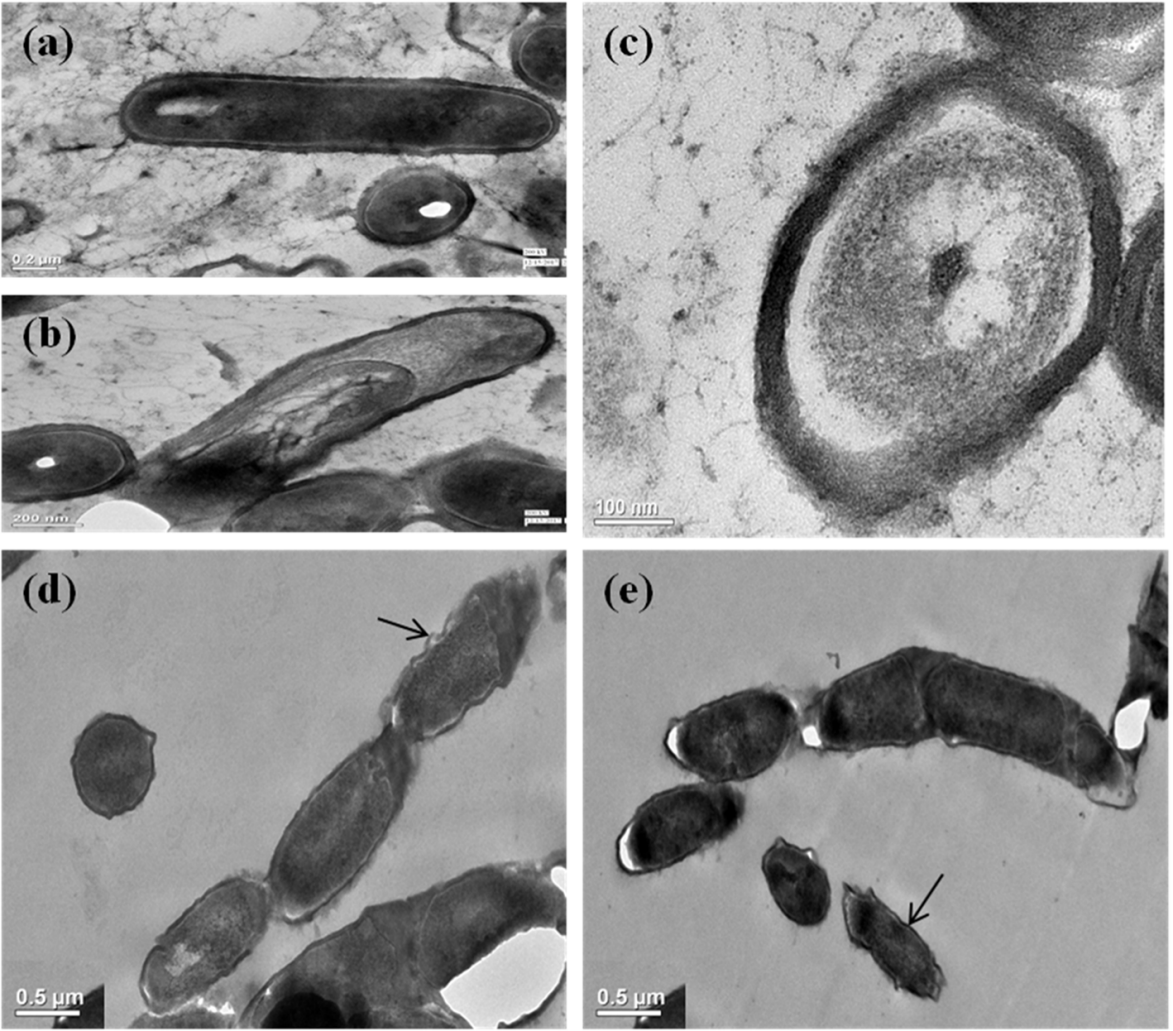
Transmission electron microscopy imaging of MHN12 cells under salt stress. **a**–**c** Longitudinal and cross section of unstressed bacterial cell (0.5%NaCl) exhibiting normal rod shape and regular cell wall. **d**, **e** Longitudinal section of stressed bacterium (15% NaCl) exhibiting incomplete cell division and irregular cell wall (indicated by arrow). *Scale bar:* **a**, **b** 0.2 μm **c** 0.1 μm. **d**, **e** 0.5 μm

As morphology of test isolate changed from bacillus to long bacterial chains, transmission electron microscopy at 0.5% salt and 15% salt was also performed. The cells growing at low salt concentration (0.5%) showed normal cell division (Fig. 6a–c). Comparison of ultra structure at these two concentrations of salt showed that the stressed cells at higher salt concentration underwent a series of morphological changes leading to filamentous morphotypes along with few normal cells (Fig. 6d, e). TEM reveals the presence of long filaments with no evident segmentation, affecting the process of cell division. In addition, deformities in cell wall were also observed (Fig. 6d).

## Discussion

The preliminary work done in our laboratory had been planned to gather the diversity of endophytic bacteria from *Vigna radiata* of different agro-climatic zones in Haryana and their potential role in crop improvement [19, 34]. In the present study, 46 endophytes isolated from nodules and roots of *V. radiata* grown in different districts were screened for their salt tolerance ability. The aim was to explore the endophytic salt tolerant plant growth promoting bacteria to be used as bioinoculant.

The selection of the isolate was based on its ability to tolerate high concentration of salt as well as plant growth promoting traits. One of the isolate MHN12 was able to withstand 15% NaCl (2.6M). In addition to this, when screened for plant growth promoting traits, isolate also exhibited traits such as ACCD, IAA, siderophore, HCN, ammonia and organic acid production. The ethylene production by plants increases in saline soil, in order to begin the programmed cell death. Some endophytic bacteria have the ability to breakdown ACC, precursor of ethylene by producing an enzyme ACC deaminase. The selected isolate had high ACCD (24.07 ± 1.79 μmoles of α-KB/mg protein/h) activity. Bacteria which produce more than 20 nmol α-K mg^−1^ protein h^-1^ can augment plant growth by decreasing levels of ethylene [35]. This was supported by study on bacterium *Arthrobacter protophormiae* which possessed ACCD activity of 241 nmol α-K mg^−1^ protein h^−1^, ameliorated the damage due to salt stress and increased the yield [36].

Indole acetic acid, an auxin is known to stimulate cell division and root initiation in plants and is an important trait of endophytic bacteria which directly influences plant growth. Previous researches carried out on role of salt tolerant PGPR producing considerable amount of IAA had reported that they alleviate the salt stress and improve the plant yield [37]. Production of siderophore by endophytes is another beneficial trait as it helps plants in uptake of various metals. Siderophores obstruct the availability of iron to phytopathogen, thus inhibiting their growth within the host plant [38]. Production of ammonia helps in plant growth and accumulation of biomass [39]. HCN, a volatile compound, reported to be produced by various endophytes, protects plants from various soil borne pathogens and help in plant growth indirectly [40]. Several studies have demonstrated that the endophytic bacteria equipped with ACCD along with other plant growth promoting attributes enhance the plant growth [41]. The presence of these multiple traits have contributed to the enhancement of all the plant growth parameters in in vivo condition as reported earlier (19). Further this halotolerant endophytic bacteria with ACCD activity and IAA production can be explored in pot and field trials under saline stress.

Phylogenetic analysis revealed similarity of MHN12 towards *Bacillus licheniformis* strain DSM 13 and *Bacillus licheniformis* strain BCRC 11702.Several studies have reported that the *Bacillus* and its derived genera are the major halotolerant bacteria isolated either from tissue or rhizosphere of plants grown in saline soils [42]. On the contrary, the isolation source of halotolerant MHN12 was from crop grown under non-saline soil. Previous work reported on *B. licheniformis* DSM 13^T^ has shown that it can bear up to 1 M NaCl salt concentrations and further rise in salinity resulted in decline in growth and at 1.3 M (7.5%) NaCl, complete inhibition of growth was observed [43]. Under high salinity, the growth of bacteria is usually inhibited due to cellular dehydration by low water activity from the environment. The growth of salt tolerant *Mesorhizobium ciceri* strain ch-191 gradually decreased with increasing salt concentration from 200 mmoll^−1^ to 400 mmol l^−1^.With increased salt concentration the duration of lag phase of cell multiplication also increased [44]. In the present study also, time period of the lag phase and log phase of growth curve increased at high salt concentrations.

Imposition of any environmental stress to microorganisms result in adaptive changes in their metabolic processes and these changes are reflected in the alteration of protein profiles [45]. The shift in banding pattern of high as well as low molecular weight polypeptides at varying salt concentrations was observed. In other reports also, increase in number of protein and inflection in existing protein was observed depicting that this alteration is responsible for survival of bacteria under stress conditions [16]. The banding pattern of 15 and 15% shock treatment were almost similar suggesting the isolate MHN12 ability to accalamatize easily to the shock treatment and no new proteins were synthesized or modulated during the log phase.

The cellular response to stress conditions is polyphasic process as it involves more than one mechanism. Studies have reported that members of *Bacillus* genera either synthesize or uptake compatible solutes such as glycine, betaine, and proline which play a significant role in their defence against high osmolarity [46, 47]. According to previous study, it has been reported that *B. licheniformis* represents a group of bacilli which synthesize proline as a dominant osmoprotectant [48]. The increase in salt concentration leads to accumulation of proline up to certain extent but at higher salt concentrations proline level declines signifying that these compounds may contribute to osmoregulation with in the tolerance range of bacteria [38]. This might be linked with the cell’s attempt to deal with bioenergetic limitation under high-salinity growth conditions or to optimize the solvent properties of the cytoplasm [49].

Morphological changes in microorganisms observed under various environmental stresses are the adaptive response of the microorganisms to cope with hostile environment and may be able to repopulate in post stress conditions [50]. Bacterium protect its cytoplasm by acquiring or synthesizing osmolytes in saline conditions but cytoplasmic membrane and cell wall are in continuous exposure to outer environment and they might undergo adaptive changes to survive [51]. Alterations in morphology of *Gluconacetobacter diazotrophicus* have also been reported in response to NH_4_Cl (ammonium chloride), NH_4_NO_3_ (ammonium nitrate) [52]; NaCl [53] and pesticides [54]. These data suggests that interference in cell wall formation occurs during cell division due to high NaCl. Formation of long filaments may be a stress marker or a type of protection mechanism to adapt till the environment recovers to normal conditions. The mechanism behind adversity adaptation as a response to salt stress, leading to an increased pleomorphic cell shape is still unclear.

Most of the plant growth promoting bacteria have difficulty surviving under stress conditions like salinity, drought and in presence of heavy metals. PGP bacterial strains have been documented to lose their potential under extreme stress conditions like salinity [55]. The response by the isolates to a long exposure of stress conditions needs to be evaluated over an extended period of time. There is still a lot to be explored at biochemical as well as molecular level that how these salt tolerant endophytes support themselves and their host under stress. An in-depth studies targeting the expressions of genes and functional potential of salt tolerant endophytes have to be conducted in future so in order to design customized bioformulations for saline soils. The exploration of this bacterium as potential bioinoculant after field experiments will surely pave a path towards clean and green environment.

## Conclusion

One of the major concern is to reduce the over usage of chemical fertilizers, and an extensive investigation towards using biofertilizers for global sustainable agriculture in saline soil would suffice as an effective strategy. Exploring natural resources to find out promising endophytes to be used as biofertilizer is much more beneficial and safer in comparison to transgenics. The current study demonstrates that halotolerant *Bacillus licheniformis* strain MHN12 possess beneficial traits and repopulate in post stress conditions. This isolate has a potential to promote plant growth in those soils where seasonal variation in salinity occurs due to climate change. Our findings of stress response (2.6 M NaCl) of *B. licheniformis* may further help in understanding the underlying molecular mechanisms for adaptation.

## Data Availability

Not applicable

## Abbreviations

ACC: 1-aminocyclopropane-1-carboxylic acid
ACCD: 1-aminocyclopropane-1-carboxylic acid deaminase
CAS: Chrome azurol S
CBB: Coomassie brilliant blue
CTAB: Hexadecyltrimethylammonium bromide
EDTA: Ethylenediamine tetra acetic acid
HCN: Hydrogen cyanide
IAA: Indole acetic acid
IMViC: Indole, Methyl red, Voges-Proskauer and Citrate utilization test
NaCl: Sodium chloride
NCBI: National Center for Biotechnology Information
NH4Cl: Ammonium chloride
NH4NO3: Ammonium nitrate
PCR: Polymerase chain reaction
SDS-PAGE: Sodium dodecyl-sulfate polyacrylamide gel electrophoresis
TBE: Tris-acetate-EDTA
TEM: Transmission electron microscopy
TSA: Tryptone soya agar
TSB: Tryptone soya broth
